# Association of the red cell distribution width/albumin ratio with 6-month readmission risk in heart failure patients

**DOI:** 10.3389/fcvm.2026.1842863

**Published:** 2026-10-15

**Authors:** Hanyu Wang, Lihua Yang, Zhengye Liu, Yitian Wang, Xing Lv, Ruina Ma, Min Li

**Affiliations:** 1 College of Basic Medicine, the Fourth Military Medical University, Xi’an, China; 2 Department of Pulmonary and Critical Care Medicine, the Second Affiliated Hospital, the Fourth Military Medical University, Xi’an, China; 3 Department of Critical Care Medicine, the Second Affiliated Hospital, the Fourth Military Medical University, Xi’an, China

**Keywords:** 6-month readmission, albumin, biomarker, heart failure, red cell distribution width

## Abstract

**Background:**

Readmission due to heart failure (HF) remains a major clinical and economic challenge. The red cell distribution width-to-albumin ratio (RAR) has been proposed as a novel biomarker reflecting chronic inflammation, nutritional status, and multi-organ involvement in cardiovascular diseases. However, the association between RAR and readmission risk, particularly in Asian populations with HF, remains unclear. This study aimed to evaluate the relationship between RAR levels and the 6-month readmission risk among Chinese patients with heart failure.

**Methods:**

We conducted a retrospective cohort study using data from the Zigong Heart Failure Database, which included 2008 patients hospitalized for HF between December 2016 and June 2019. After excluding patients with missing RDW, albumin, or readmission data, 1854 patients were included in the final analytical cohort. Based on RAR values, patients were divided into four quartiles. Multivariable Cox proportional hazards regression models were used to assess the association between RAR and 6-month readmission risk, adjusting for demographics, clinical parameters, comorbidities, and medications. Subgroup analyses were performed to examine the consistency of associations across different patient characteristics.

**Results:**

The overall 6-month readmission rate was 38.5% (714/1854). Patients in higher RAR quartiles had a significantly greater risk of readmission than those in lower quartiles (*P* = 0.0025). In unadjusted analyses, each unit increase in RAR was associated with a 57% higher risk of 6-month readmission (HR 1.57, 95% CI: 1.27–1.93, *P* < 0.001). This association remained significant after adjusting for confounding variables (HR 1.52, 95% CI: 1.21–1.92, *P* < 0.001). After full adjustment, patients in the highest quartile (Q4) had a 39% higher risk of readmission compared with those in the lowest quartile (Q1) (HR 1.39, 95% CI: 1.10–1.76, *P* < 0.001). Subgroup analyses showed consistent associations across sex, age, BMI, NYHA classification, and medication use, with no significant interactions observed.

**Conclusion:**

Elevated RAR was independently associated with an increased 6-month readmission risk among Chinese patients with HF. As a simple and cost-effective biomarker, RAR may enhance risk stratification and inform clinical decision-making in HF management, potentially improving patient outcomes and reducing healthcare burden.

## Introduction

The prevalence of heart failure (HF) stems from an aging population and modern therapeutic advances that have prolonged the lives of patients with cardiovascular disease (1, 2). HF remains a major cause of hospitalization in both the United States and Europe (3). In the United States alone, approximately one million HF-related admissions and readmissions occur annually (4). Furthermore, 20%–50% of patients discharged afterhospitalization for HF are readmitted within 30 days to one year (5). The substantial costs associated with HF management impose a considerable financial burden on patients, potentially compromising adherence to guideline-directed medical therapies. Readmission serves as a critical clinical indicator of HF severity and contributes significantly to patient prognosis, while also imposing major economic and public health burdens (6, 7). Red cell distribution width (RDW) measures the variability in the size of circulating red blood cells. Its clinical relevance in patients with chronic heart failure (CHF) has been increasingly recognized (8). Moreover, RDW has been suggested to reflect chronic low-grade inflammation in the body (9, 10). Human serum albumin (HSA) possesses multiple physiological functions—including anti-inflammatory, antioxidant, anticoagulant, and antiplatelet therapy effects—making it a key indicator of nutritional and inflammatory status (11). The RDW-to-albumin ratio (RAR) is a composite biomarker that comprehensively reflects multidimensional physiological dysfunction, including systemic inflammatory dysfunction, nutritional status, and aging. Its predictive value may surpass that of either individual indicator. Because chronic low-grade inflammation, aging, and malnutrition are major factors contributing to readmission risk among patients with CHF (12), RAR may represent a promising biomarker for predicting such risk. However, no prior study has evaluated the association between RAR and readmission among patients with HF, particularly within an Asian population. Therefore, this study aimed to assess the association between RAR levels and 6-month readmission risk in Chinese patients with CHF.

## Methods

### Data source

This study employed a retrospective cohort design. Relevant data were extracted from an online open-source database of Chinese patients with HF, accessible via PhysioNet (https://physionet.org/content/heart-failure-zigong/1.2/) (13). This database contains continuous retrospective collections of electronic medical records from 2008 patients with HF hospitalized at Zigong Fourth People's Hospital between December 2016 and June 2019. Patients with HF were identified using the International Classification of Diseases (ICD)-9 coding system (14). The diagnosis of HF was made in accordance with the 2016 guidelines of the European Society of Cardiology (ESC) (15). The database, which included human participants, was constructed in compliance with the Declaration of Helsinki and its subsequent amendments. The research protocol and methodologies employed in this study were approved by the Ethics Committee of Zigong Fourth People's Hospital (Approval No. 2020-010). Given the retrospective nature of this study, the requirement for informed consent was waived by the Ethics Committee.

### Study design and population

This study recruited hospitalized patients with chronic heart failure (NYHA Class II–IV). The exclusion criteria were as follows: (1) missing red cell distribution width data, (2) missing serum albumin data, and (3) absence of readmission time data. Clinical characteristics and medical histories were collected upon admission, including demographic and vital sign parameters such as age, sex, body mass index (BMI), New York Heart Association (NYHA) cardiac function classification, Heart failure classification, HF etiology, All-cause readmission within six months, systolic blood pressure (SBP), diastolic blood pressure (DBP), body temperature, pulse rate, and respiratory rate. Comorbidities included a history of myocardial infarction (MI), chronic obstructive pulmonary disease (COPD), diabetes, chronic kidney disease (CKD), liver disease, and cerebrovascular disease.

Within the first 24 h of admission, laboratory test results were obtained, including red cell distribution width (RDW), albumin, c reactive protein(CRP), serum creatinine, urea, uric acid, white blood cell count, hemoglobin, platelet count, D-dimer, calcium, potassium, chloride, sodium, total cholesterol, brain natriuretic peptide (BNP), lactate, low-density lipoprotein cholesterol (LDL-C), high-density lipoprotein cholesterol (HDL-C), triglycerides, estimated glomerular filtration rate (eGFR), high-sensitivity cardiac troponin (hs-cTn), potential of hydrogen (pH), partial pressure of carbon dioxide (PaCO₂), and partial pressure of oxygen (PaO₂). Medications administered during hospitalization—including renin–angiotensin–aldosterone system (RAAS) inhibitors, beta-blockers, diuretics, antiplatelet therapy, anticoagulants, statins, and inotropic agents—were also meticulously recorded.

### Outcome

The main outcome was all-cause readmission within six months calculated from the date of index hospital admission. All-cause readmission was defined as readmission for any cause after discharge for heart failure. Data on hospital admissions within six months were obtained at a mandatory follow-up visit or via telephone call.

### Covariate

Variables that may be related to RAR and readmission should be adjusted for to increase the reliability of the results. This mainly included demographic data (age, gender and BMI), cardiac function (NYHA classification, hs-Troponin, BNP),clinical co-morbidities [Charlson Comorbidity Index score(CCI score)], cardiac medication (antiplatelet therapy use, anticoagulation use, ACEI/ARB use, *β*-blocker use, inotropic agents use, diuretic use, statin use).

### Statistical analysis

The RAR was divided into four groups based on quartiles. Descriptive statistics were used to characterize the fundamental features of the four groups. Continuous variables with a normal distribution were described using mean and standard deviation (SD), whereas the median and interquartile range (IQR) were used for data that were not normally distributed. Categorical variables were presented as proportions. One-way analysis of variance (for normal distributions), chi-square tests (for categorical variables), and Kruskal–Wallis tests (for skewed distributions) were used to assess differences among the four groups. In addition, missing data were handled by multiple imputation.

To assess the relationship between RAR and 6-month readmission risk, multivariable Cox proportional hazards regression analyses were performed. The crude model was unadjusted. Model 1 was adjusted for age and sex. Model 2 was expanded upon Model 1 by incorporating BMI, NYHA classification, hs-troponin levels, BNP levels, and CRP levels. Model 3 included all variables from Model 2 and the CCI score. Model 4 was adjusted based on Model 3 with the addition of antiplatelet therapy use, anticoagulation use, ACEI/ARB use, *β*-blocker use, inotropic agents use, diuretic use, and statin use. The Kaplan–Meier analysis was used to estimate cumulative readmission rates over time. Differences between the high and low RAR groups were assessed using the log-rank test. Finally, subgroup and interaction analyses were performed to identify potential modifiers according to sex, age (<70 vs. ≥ 70 years), gender (male vs. female), BMI (BMI <21, 21 ≤ BMI <25, BMI ≥25 kg/m^2^), NYHA class (II, III, IV), inotropic agents use, diuretic use, *β*-blocker use, statin use, and ACEI/ARB use. The results were visualized as a forest plot. Analyses were performed using R version 4.2.2 (http://www.R-project.org; The R Foundation, Vienna, Austria) and Free Statistics software (version 2.2; Beijing FreeClinical Medical Technology Co., Ltd., Beijing, China).

## Results

### Population characteristics

A total of 2008 patients diagnosed with HF were enrolled in this retrospective study. After excluding 132 patients with missing red blood cell distribution width and albumin values at admission and 22 patients lacking readmission time data, the final analytical dataset consisted of 1,854 patients (Figure 1).

**Figure 1:**
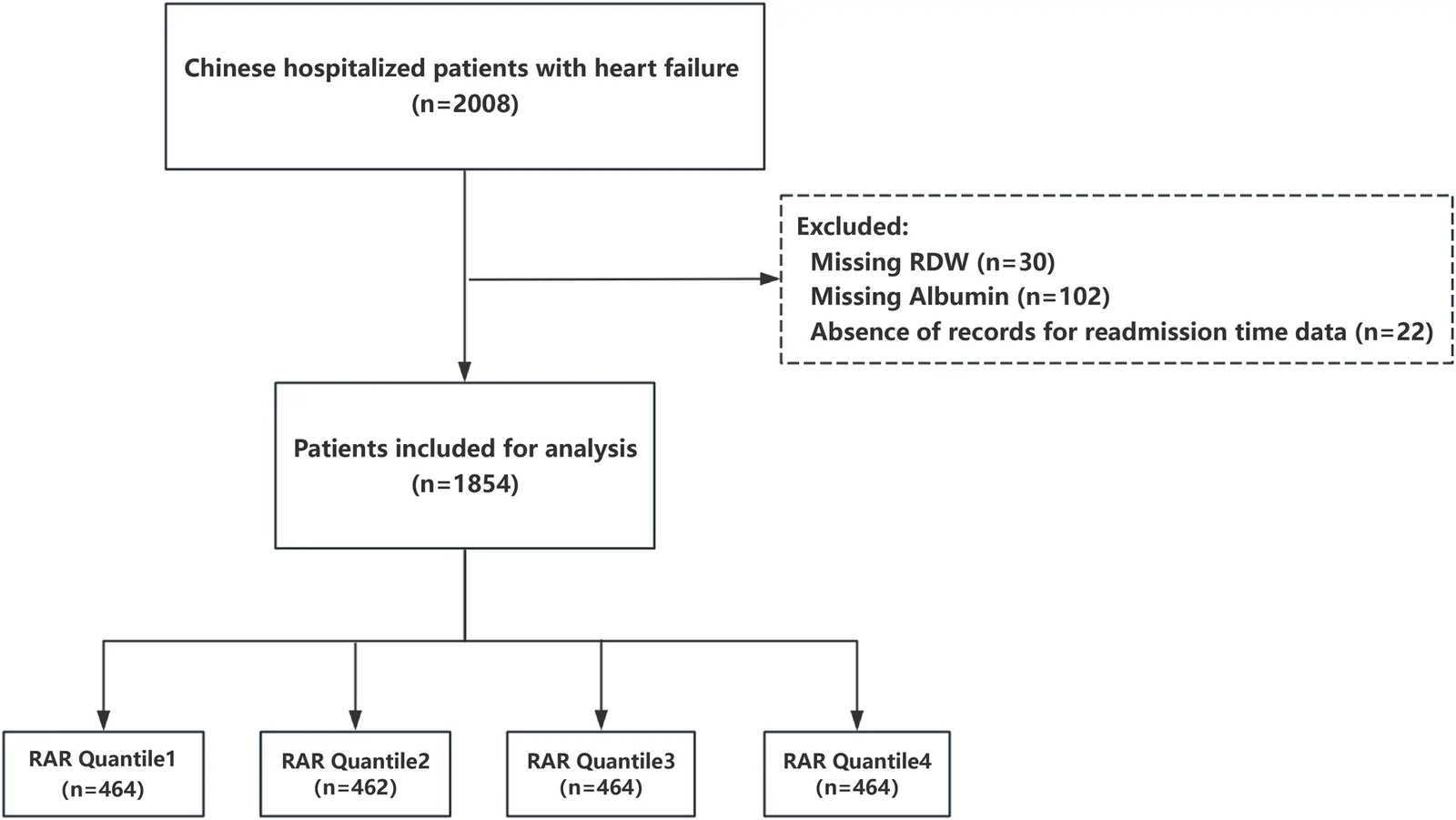
The flowchart of patients’ inclusion and exclusion.

The total readmission rate over six months was 38.5% (714/1,854 patients). Among these participants, individuals aged over 70 years constituted 73.4% of the cohort. Compared to patients in the low-RAR group (Quartile 1 and Quartile 2), male patients in the high-RAR group (Quartile 3 and Quartile 4) exhibited poorer cardiac function, whereas female patients in the high-RAR group demonstrated better cardiac function. Most patients presented with cardiac function classified as Class III or IV in the entire population, and they also displayed elevated levels of various blood biomarkers (Table 1). Furthermore, nearly all patients received diuretic therapy. The baseline clinical characteristics are detailed in Table 1.

**Table 1:** Baseline characteristics of the patients.

	RAR					
Variables	Total(*n* = 1,854)	Quartile1 (*n* = 464)	Quartile2 (*n* = 462)	Quartile3 (*n* = 464)	Quartile4 (*n* = 464)	*P*
Age,years, n(%)						< 0.001
<70	494 (26.6)	176 (37.9)	121 (26.2)	101 (21.8)	96 (20.7)	
≥70	1,360 (73.4)	288 (62.1)	341 (73.8)	363 (78.2)	368 (79.3)	
Gender						0.001
Female	1,078 (58.1)	295 (63.6)	283 (61.3)	259 (55.8)	241 (51.9)	
Male	776 (41.9)	169 (36.4)	179 (38.7)	205 (44.2)	223 (48.1)	
BMI(kg/m^2^)	21.3 ± 3.8	21.8 ± 3.8	21.6 ± 4.0	20.7 ± 3.6	21.1 ± 3.8	< 0.001
NYHA cardiac function classification						< 0.001
II	318 (17.2)	98 (21.1)	81 (17.5)	76 (16.4)	63 (13.6)	
III	962 (51.9)	264 (56.9)	249 (53.9)	224 (48.3)	225 (48.5)	
IV	574 (31.0)	102 (22)	132 (28.6)	164 (35.3)	176 (37.9)	
Heart failure classification						0.678
HFrEF	137 (7.4)	36 (7.8)	40 (8.7)	35 (7.5)	26 (5.6)	
HFmrEF	127 (6.9)	33 (7.1)	29 (6.3)	33 (7.1)	32 (6.9)	
HFpEF	324 (17.5)	92 (19.8)	75 (16.2)	75 (16.2)	82 (17.7)	
Unclassified	1,266 (68.3)	303 (65.3)	318 (68.8)	321 (69.2)	324 (69.8)	
HF etiology						0.214
Ischemic	133 (7.2)	26 (5.6)	39 (8.4)	29 (6.2)	39 (8.4)	
Non-ischemic	1,721 (92.8)	438 (94.4)	423 (91.6)	435 (93.8)	425 (91.6)	
SBP (mmHg)	131.3 ± 24.6	132.9 ± 25.3	133.0 ± 24.0	131.4 ± 23.4	127.9 ± 25.5	0.005
DBP (mmHg)	76.6 ± 14.5	78.1 ± 14.5	77.6 ± 14.1	76.9 ± 14.7	73.9 ± 14.4	< 0.001
Body temperatures(℃)	36.4 ± 0.4	36.4 ± 0.4	36.4 ± 0.5	36.4 ± 0.4	36.4 ± 0.5	0.549
Pulse(b.p.m.)	85.3 ± 21.5	85.2 ± 20.1	86.3 ± 21.3	85.5 ± 21.7	84.4 ± 22.8	0.615
Respiration	19.1 ± 1.8	19.0 ± 1.4	19.2 ± 2.0	19.0 ± 1.5	19.2 ± 2.0	0.079
Myocardial infarction	133 (7.2)	26 (5.6)	39 (8.4)	29 (6.2)	39 (8.4)	0.214
COPD	221 (11.9)	47 (10.1)	54 (11.7)	57 (12.3)	63 (13.6)	0.438
Diabetes	435 (23.5)	117 (25.2)	90 (19.5)	103 (22.2)	125 (26.9)	0.038
CKD	439 (23.7)	89 (19.2)	87 (18.9)	120 (25.9)	143 (30.8)	< 0.001
Liver disease	78 (4.2)	13 (2.8)	17 (3.7)	19 (4.1)	29 (6.3)	0.059
CVD	136 (7.3)	29 (6.2)	34 (7.4)	37 (8)	36 (7.8)	0.752
RDW(fL)	47.9 (45.2, 51.4)	44.4 (42.3, 46.5)	46.8 (45.2, 48.7)	49.5 (46.8, 52.0)	53.3 (49.9, 57.9)	< 0.001
Albumin (g/dL)	36.5 ± 5.0	41.5 ± 3.3	37.9 ± 2.5	35.6 ± 2.8	31.1 ± 4.1	< 0.001
CRP(Mean ± SD)	25.3 ± 34.9	18.2 ± 28.5	20.7 ± 30.7	25.3 ± 34.7	35.2 ± 40.9	< 0.001
Serum creatinine (*μ*mol/L)	87.6 (65.2, 123.9)	78.4 (60.7, 105.8)	83.8 (64.7, 113.0)	91.6 (68.9, 130.3)	102.0 (70.3, 149.0)	< 0.001
Urea (mmol/L)	8.1 (5.9, 11.6)	7.1 (5.5, 9.6)	7.5 (5.7, 10.7)	8.3 (6.0, 12.2)	9.8 (6.7, 14.3)	< 0.001
Uric acid (μmol/L)	483.7 ± 168.5	458.7 ± 146.8	468.3 ± 164.8	491.9 ± 179.0	516.0 ± 176.2	< 0.001
White blood cell(10^9^/L)	7.3 ± 3.5	7.6 ± 3.2	7.3 ± 3.1	7.2 ± 3.6	7.2 ± 3.9	0.193
Hemoglobin (g/L)	114.9 ± 24.3	123.2 ± 21.3	119.3 ± 20.5	113.6 ± 23.0	103.5 ± 27.1	< 0.001
Platelet (10^9^/L)	144.5 ± 64.3	148.9 ± 56.5	145.4 ± 63.2	139.5 ± 58.4	144.3 ± 76.9	0.167
D.dimer(mg/L)	1.2 (0.8, 2.2)	0.9 (0.7, 1.3)	1.1 (0.8, 1.8)	1.4 (0.9, 2.3)	1.9 (1.2, 4.2)	< 0.001
Calcium (mmol/L)	2.3 ± 0.2	2.4 ± 0.2	2.3 ± 0.1	2.3 ± 0.2	2.2 ± 0.2	< 0.001
Potassium (mmol/L)	4.0 ± 0.7	3.9 ± 0.8	3.9 ± 0.6	4.0 ± 0.7	4.0 ± 0.7	0.034
Chloride (mmol/L)	101.8 ± 6.0	101.4 ± 5.8	102.1 ± 5.6	102.0 ± 6.1	101.9 ± 6.6	0.284
Sodium (mmol/L)	138.3 ± 4.9	138.4 ± 5.1	138.8 ± 4.6	138.2 ± 5.1	137.7 ± 4.8	0.018
Cholesterol (mmol/L)	3.7 ± 1.1	4.1 ± 1.1	3.9 ± 1.0	3.6 ± 1.0	3.3 ± 1.1	< 0.001
BNP (pg/mL)	1,299.7 ± 1,354.0	838.4 ± 1,059.0	1,279.9 ± 1,382.4	1,382.4 ± 1,307.0	1,702.9 ± 1,493.0	< 0.001
Lactate (mmol/L)	1.8 (1.5, 2.5)	1.8 (1.5, 2.4)	1.8 (1.5, 2.3)	1.9 (1.5, 2.6)	1.8 (1.4, 2.5)	0.569
LDL-C (mmol/L)	1.9 ± 0.8	2.1 ± 0.8	2.0 ± 0.8	1.8 ± 0.7	1.6 ± 0.7	< 0.001
HDL-C (mmol/L)	1.1 ± 0.4	1.2 ± 0.4	1.1 ± 0.3	1.1 ± 0.3	1.0 ± 0.4	< 0.001
Triglyceride (mmol/L)	1.0 (0.7, 1.3)	1.1 (0.8, 1.5)	1.0 (0.8, 1.3)	0.9 (0.7, 1.2)	0.8 (0.7, 1.1)	< 0.001
eGFR (mL/min/1.73 m^2^)	67.9 ± 36.2	76.8 ± 35.8	70.9 ± 35.2	63.5 ± 32.5	60.5 ± 38.7	< 0.001
Hs-cTn (pg/mL)	0.3 ± 2.0	0.2 ± 1.3	0.3 ± 2.1	0.3 ± 1.7	0.4 ± 2.7	0.279
pH	7.4 ± 0.1	7.4 ± 0.1	7.4 ± 0.1	7.4 ± 0.1	7.4 ± 0.1	0.687
PaO_2_ (mmHg)	108.1 ± 37.4	106.4 ± 33.2	108.8 ± 39.1	108.6 ± 38.8	108.3 ± 38.0	0.891
PaCO_2_ (mmHg)	35.8 ± 9.7	35.6 ± 7.1	35.4 ± 10.2	35.3 ± 9.5	36.7 ± 11.1	0.374
ACEI.ARB	717 (38.7)	212 (45.7)	187 (40.5)	171 (36.9)	147 (31.7)	< 0.001
Beta blocker	692 (37.3)	198 (42.7)	197 (42.6)	167 (36)	130 (28)	< 0.001
Diuretics	1,825 (98.4)	449 (96.8)	459 (99.4)	456 (98.3)	461 (99.4)	0.004
Antiplatelet therapy	1,121 (60.5)	259 (55.8)	282 (61)	289 (62.3)	291 (62.7)	0.118
Anticoagulation	362 (19.5)	114 (24.6)	88 (19)	91 (19.6)	69 (14.9)	0.003
Statin use	764 (41.2)	175 (37.7)	180 (39)	215 (46.3)	194 (41.8)	0.038
Inotropic agents	939 (50.6)	234 (50.4)	243 (52.6)	242 (52.2)	220 (47.4)	0.38

Data are presented as mean ± SD, median (Interquartile range) or *n* (%).

BMI, body mass index; NYHA, New York Heart Association; SBP, systolic blood pressure; DBP, diastolic blood pressure; CVD, Cerebrovascular disease; COPD, chronic obstructive pulmonary disease; CKD, chronic kidney disease; LDL-C, low-density lipoprotein cholesterol; HDL-C, high-density lipoprotein cholesterol; eGFR, estimated glomerular filtration rate; hs-cTn, high sensitivity cardiac troponin; BNP, brain natriuretic peptide; pH, potential of hydrogen; PaCO2, partial pressure of carbon dioxide; PaO2, partial pressure of oxygen; SD, standard deviation.

### Association between the RAR and the risk of 6-month readmission

A greater RAR was linked to a higher probability of 6-month readmission in multivariable Cox regression analysis (Table 2). In the Crude model, there was a 57% rise in the likelihood of the adverse outcome for every 1 unit increase in RAR (hazard ratio [HR] 1.57, 95% confidence interval [CI]: 1.27-1.93, *P* ＜ 0.001; Table 2). After adjusting for confounding variables, the results were obtained. (HR 1.52, 95% Cl: 1.21-1.92, *P* ＜ 0.001; Table 2, model 4). Patients in the highest RAR group (Q4) had a 39% higher risk of 6-month readmission compared to patients in the lowest RAR group (Q1) after correcting for potential confounding variables (HR 1.39, 95% CI: 1.10-1.76, *P* ＜ 0.001; Table 2, model 4). The cumulative incidence of 6-month readmission, illustrated in Figure 2, indicated that patients with a high RAR level had a significantly elevated risk of 6-month readmission compared to those with a low RAR level (log-rank *P* = 0.0025). Kaplan–Meier curves for 6-month readmission rates stratified by RAR level. The high RAR group exhibited a significantly higher readmission rate than the low RAR group (log-rank *P* = 0.0025).

**Table 2:** Multivariable analysis of RAR with 6-month readmission in Chinese adult patients with heart failure.

Models	Overall group		Quartile groups			
	（*n* = 1,854)		Q1（*n* = 464)	Q2（*n* = 462)	Q3（*n* = 464)	Q4（*n* = 464)
	HR（95%CI）	*P*	HR（95%CI）	HR（95%CI）	HR（95%CI）	HR（95%CI）
Crude model	1.57 (1.27∼1.93)	<0.001	1(Ref)	1.12 (0.91∼1.38)	1.24 (1.01∼1.54)	1.46 (1.18∼1.8)
Mode1 1	1.54 (1.24∼1.9)	<0.001	1(Ref)	1.09 (0.88∼1.35)	1.22 (0.98∼1.51)	1.42 (1.15∼1.76)
Mode1 2	1.45 (1.16∼1.82)	<0.001	1(Ref)	1.06 (0.86∼1.31)	1.12 (0.89∼1.39)	1.33 (1.06∼1.66)
Mode1 3	1.46 (1.16∼1.84)	<0.001	1(Ref)	1.1 (0.88∼1.36)	1.11 (0.89∼1.38)	1.33 (1.06∼1.67)
Mode1 4	1.52 (1.21∼1.92)	<0.001	1(Ref)	1.12 (0.9∼1.39)	1.12 (0.89∼1.4)	1.39 (1.1∼1.76)

Mode1 1 was adjusted by age, sex.

Model 2 was adjusted by Model 1 plus BMI, NYHA classification, hs-Troponin, BNP,CRP.

Model 3 was adjusted by Model 2 plus CCI score.

Model 4 was adjusted by Model 3 plus antiplatelet therapy use,anticoagulation use, ACEI/ARB use, *β*-blocker use,inotropic agents use, diuretic use, statin use.

RAR, the red cell distribution width/albumin ratio; Q1, Quartile1; Q2, Quartile2; Q3, Quartile3; Q4, Quartile4; HR, hazard ratio; CI, confidence interval.

.

**Figure 2:**
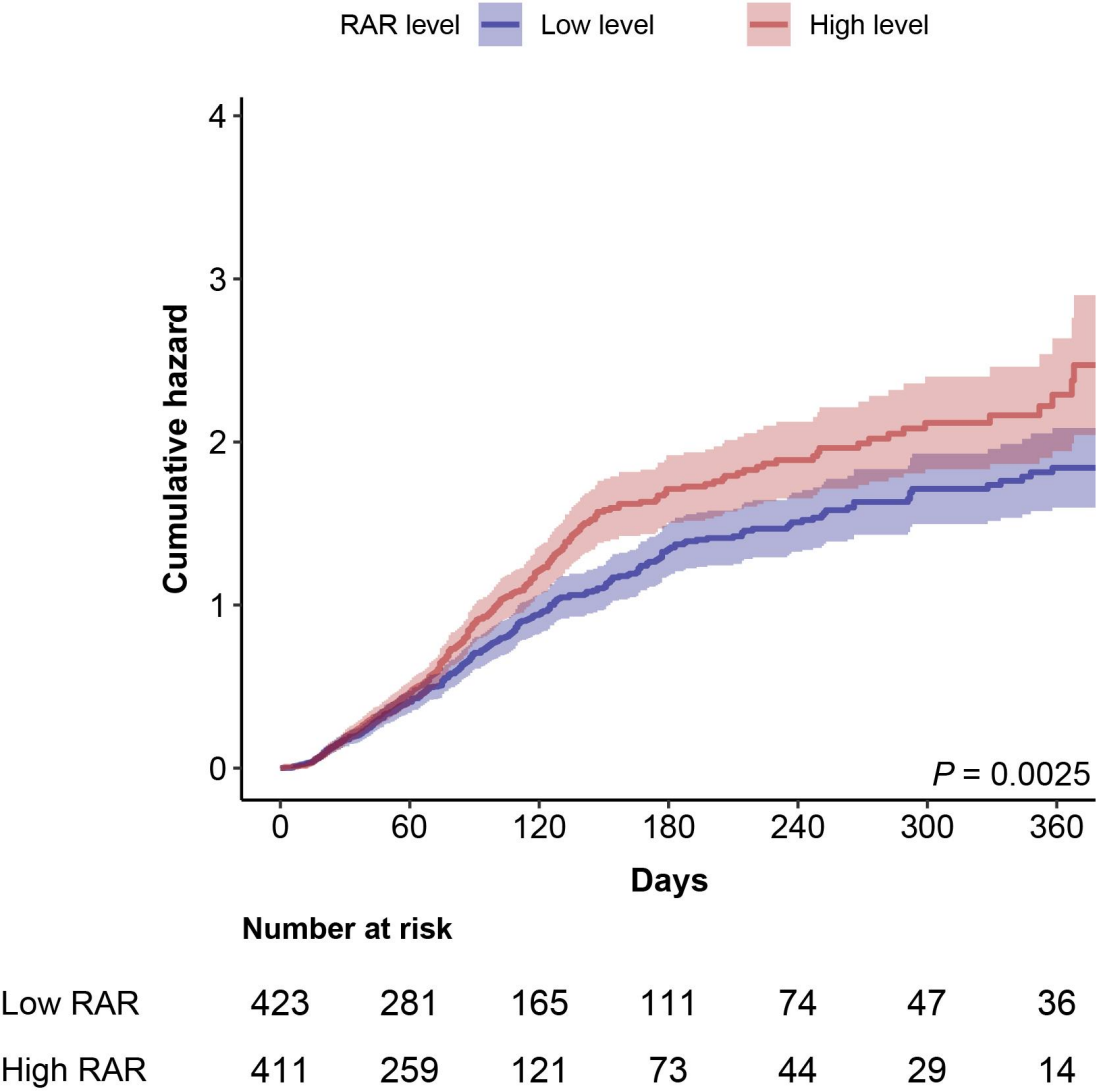
Cumulative hazard of 6-month readmission according to RAR level. RAR, the red cell distribution width/albumin ratio.

### Subgroup and sensitivity analyses

We performed subgroup and interaction analyses to ascertain the potential association between RAR and the likelihood of 6-month readmission (Figure 3). Consistent findings were observed when the study was stratified by sex, age, BMI, NYHA class, heart failure type, inotropic agents use, ACEI/ARB use, *β*-blocker use, and statin use. No significant interactions were observed. This stable association highlights the independent predictive value of RAR for 6-month readmission.

**Figure 3:**
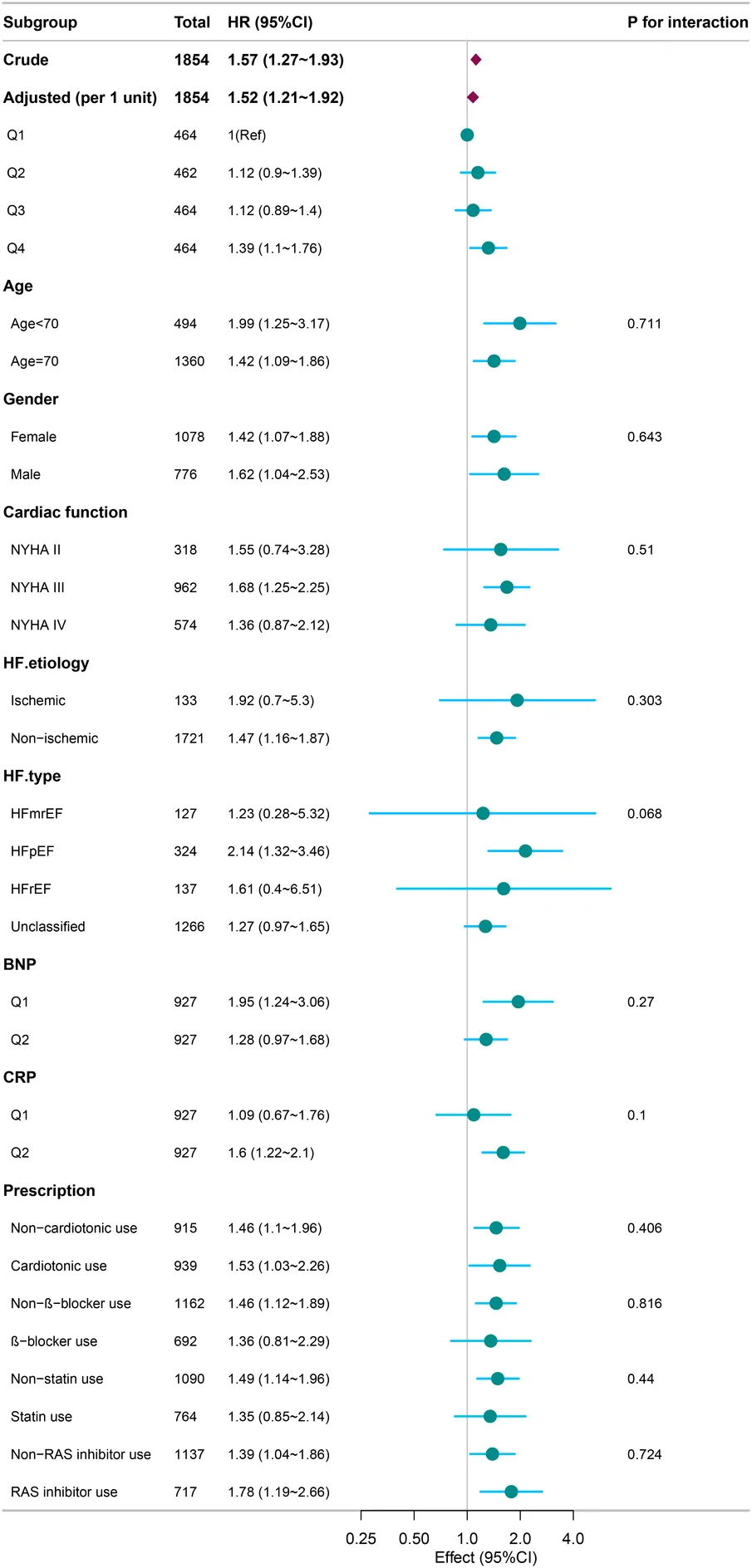
Forest plot for subgroup analysis of the relationship between RAR and 6-month readmission in Chinese HF patients. BMI, body mass index; NYHA, New York Heart Association Functional Classification; Q1, Quartile1; Q2, Quartile2; Q3, Quartile3; Q4, Quartile4; HR, hazard ratio; CI, confidence interval.

## Discussion

To examine the association between RAR and the risk of readmission within six months, we conducted a retrospective cohort study comprising 1,854 patients. Our analysis identified a significant positive linear relationship between RAR and the likelihood of readmission within a six-month period. Specifically, patients with an elevated RAR demonstrated a 39% increased risk of readmission at six months compared with those with a lower RAR after adjusting for potential confounders. This association remained consistent across subgroup analyses, with no significant interaction effects observed. RAR, proposed as a novel biomarker indicative of inflammation and nutritional status, may play a crucial role in the management of discharged patients with HF. Readmission rates are of significant value in the management of patients with HF and in assessing their prognosis. Previous studies have examined the correlation between RAR and mortality in various populations and found that RAR is positively correlated with mortality, demonstrating good predictive value in patients with heart failure (16), acute myocardial infarction (17), atrial fibrillation (18), diabetes mellitus (19) and chronic kidney disease (20). Under normal circumstances, patients with a simultaneously higher risk of short- and long-term mortality also have an increased risk of readmission. In the population analyzed, the proportion of patients with both diabetes and chronic kidney disease exceeded 25%. In addition, Zhou et al. (21) found that RAR exhibited good performance in models predicting mortality and heart transplantation in patients with CHF. As these events are closely associated with readmission, RAR is significantly correlated with readmission in patients with CHF.

Gu et al. (22) included 1,878 patients with HF in their study and found that a higher RDW value was significantly associated with the risk of 3-month readmission among hospitalized patients with HF. Compared with that study, our research also considered inflammatory factors represented by the HSA indicator, and the longer readmission period better reflected the impact of chronic inflammation on patients over time. Lin et al. (23) conducted a study of 1,978 patients with HF and found that the red blood cell distribution width-to-platelet ratio (RPR) had no significant impact on 6-month readmission (OR = 1.073, 95% CI: 0.991–1.161). A plausible explanation for this outcome is the limited number of factors considered in their analysis. In comparison, our study incorporated a broader range of variables, including comorbidities and an expanded set of laboratory parameters.

An increase in RAR may be associated with both high RDW and low HSA. The underlying biological mechanisms may be related to chronic inflammation, iron mobilization disorders, and nutritional status. Higher RDW is associated with more severe chronic inflammation and biochemical evidence of iron mobilization disorders (24). RDW may be involved in the molecular pathways of chronic inflammation and, together with other biomarkers related to immune lipids, may interfere with metabolic processes in patients with chronic heart failure (25–27). Moreover, RDW may also be linked to iron mobilization disorder, which can inhibit erythrocyte maturation induced by erythropoietin and lead to anemia of chronic disease (28). Therefore, elevated RDW may serve as a comprehensive indicator of several important pathophysiological processes in heart failure (29, 30). Serum albumin concentration is an important marker of nutritional status and inflammatory response (11, 31). In cardiovascular diseases, the underlying mechanisms are strongly associated with chronic inflammation and nutritional factors. Thus, we hypothesized that the integration of these two biomarkers could comprehensively reflect inflammation, nutrition, and related abnormalities. As a biomarker derived from routine laboratory tests, RAR may be clinically useful for evaluating readmission risk in patients with heart failure. This study has several advantages. First, we selected a cohort of Chinese patients with heart failure, and the results remained robust even after conducting various sensitivity analyses. Second, the RAR indicator is simple, easy to obtain, and can serve as an important routine laboratory biomarkers. Whether RAR is associated with the development of heart failure in broader populations will be the focus of our future research. However, this study also has certain limitations. First, as a retrospective study, the relationship between RAR and 6-month readmission indicates correlation rather than causation. Second, RAR was evaluated only at a single time point, and dynamic changes or potential trajectories were not considered. Third, although we adjusted for numerous potential confounding factors—including demographic variables, comorbidities, and medication use—we cannot exclude the possibility of residual confounding. Finally, because the database lacked detailed etiology of readmission, our primary outcome reflects all-cause rehospitalization rather than heart failure-specific readmission, which may introduce heterogeneity in the endpoint.

## Conclusion

Our study of Chinese patients with heart failure (HF) demonstrated a positive association between RAR and the risk of 6-month readmission. These findings suggest that the RAR may serve as a predictor of readmission during the post-discharge vulnerability period in this population. This study addresses the paucity of data on the prognostic utility of RAR in HF, particularly in East Asian cohorts.

## Data Availability

The datasets presented in this study can be found in online repositories. The names of the repository/repositories and accession number(s) can be found below: https://www.physionet.org/content/heart-failure-zigong/1.2/.
